# Bioassay of the infectivity of heat-treated *Toxoplasma gondii* cysts in susceptible C57BL/6J mice

**DOI:** 10.1016/j.fawpar.2026.e00315

**Published:** 2026-01-14

**Authors:** Zhao Li, Tao Li, Lian-Tao Yang, Cai-Qin Deng, Qi-Xin Liu, Qin-Zhang, Ling Wu, Yue Sun, Feng-Cai Zou, Xue Zhou, Qi-Shuai Liu

**Affiliations:** a Animal Research and Resource Center, School of Life Sciences, Yunnan University, Kunming 650500, China; b Division of Pathobiology, Faculty of Veterinary Medicine, Khon Kaen University, Khon Kaen 40002, Thailand; c The Yunnan Key Laboratory of Veterinary Etiological Biology, College of Veterinary Medicine, Yunnan Agricultural University, Kunming 650201, China

**Keywords:** *Toxoplasma gondii*, Cysts, Heat-inactivated, Bioassay, Infectivity

## Abstract

*Toxoplasma gondii* is a significant foodborne parasite. However, the precise thermal conditions required to inactivate its tissue cysts in meat remain poorly defined. This study systematically determined the effects of temperature (45–70 °C) and time (10–30 min) on cyst viability. Cysts treated under each condition were orally administered to susceptible C57BL/6J mice, and infectivity was comprehensively assessed through survival, clinical signs, serology (IgG), qPCR, and histopathology. Results demonstrated that treatment at 60 °C for 10 min or under more stringent conditions completely abolished infectivity, as evidenced by 100% survival, the absence of specific antibodies, and the non-detection of parasite DNA or lesions in tissues. Thus, 60 °C for 10 min is established as a critical inactivation threshold, providing a definitive reference for developing science-based thermal processing guidelines to enhance meat safety.

## Highlights

• Establishes 60 °C for 10 min as the thermal threshold to inactivate *T. gondii* cysts.
• A multi-parameter assessment confirms complete loss of cyst infectivity.
• Oral infection in susceptible mice provides relevant data for food safety.
• Findings directly inform thermal processing guidelines for meat safety.

## Graphical abstract

**Figure:**
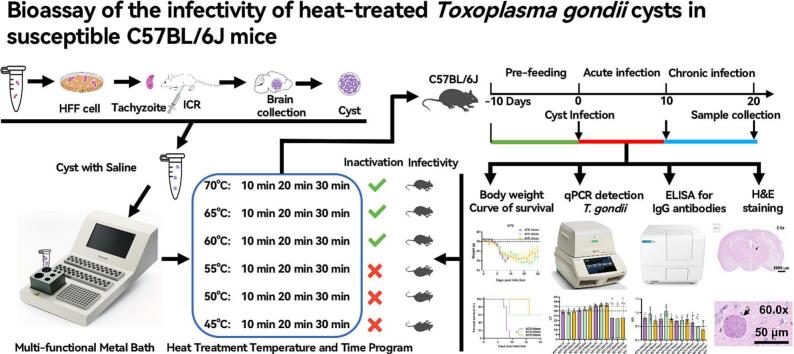

## 1 Introduction

*Toxoplasma gondii* is a cosmopolitan zoonotic parasite with a complex life cycle that encompasses sexual reproduction in felines and asexual proliferation in various intermediate hosts, including humans (Sheffield and Melton, 1970; Dubey et al., 1998; Dubey, 2022). It infects a broad spectrum of warm-blooded animals, with approximately one-third of the global human population seropositive and rates exceeding 68% in certain endemic regions (Sengupta et al., 2025). Clinical outcomes depend on host immune status, ranging from latent infection in immunocompetent individuals to severe opportunistic disease in immunocompromised individuals and congenital toxoplasmosis resulting from prenatal transmission (Chang et al., 2023; Elsheikha et al., 2020; Matta et al., 2021; Nguyen et al., 2023; Rostami et al., 2020).

Human infection occurs mainly through the foodborne route (>60% of cases), primarily via consumption of raw or undercooked meat containing tissue cysts (Cook et al., 2000; Deng et al., 2020; Ducrocq et al., 2021; Sadeghi et al., 2021; Wang et al., 2012), including game meat (Amouei et al., 2025). Despite the influence of processing techniques (e.g., freezing, salting, and curing) on cyst viability (Mirza Alizadeh et al., 2018; Rani and Pradhan, 2021), thermal cooking remains critical, with temperature and time serving as the key determinants of inactivation (Guo et al., 2015). Current prevention focuses on high-risk groups, prenatal screening, and food safety controls (Marin-Garcia et al., 2022). However, systematic studies defining precise thermal inactivation parameters are constrained by methodological inconsistencies and simplistic infectivity assessments (Dubey et al., 1990; Opsteegh et al., 2024), which underscores the necessity for a comprehensive, multi-parameter evaluation.

This study aimed to determine the critical temperature-time combination required to inactivate *T. gondii* cysts. Experimental gradients were established, ranging from 45 to 70 °C (5 °C increments) and 10–30 min (10-min increments). Cysts were administered orally to susceptible C57BL/6J mice for each treatment (Li et al., 2025). Infectivity was comprehensively assessed using a panel of metrics: clinical monitoring, survival analysis, qPCR for parasite DNA, ELISA for *T. gondii*-specific IgG antibodies, and histopathological examination. By integrating these results, this study aims to provide a definitive reference for thermal inactivation protocols in meat processing and to develop a reliable bioassay for assessing cyst viability, thereby contributing to improved food safety measures (Marin-Garcia et al., 2022).

## 2 Materials and methods

### 2.1 Parasite strain, culture, and purification

Tachyzoites of the *T. gondii* ME49 strain were obtained from Yunnan University, where they had been cryopreserved in liquid nitrogen. For expansion, the parasites were cultured at 37 °C under 5% CO₂ in confluent monolayers of human foreskin fibroblast (HFF) cells (Wang et al., 2024). Tachyzoites were harvested from heavily infected HFF cultures by mechanical disruption using a 27-gauge needle. Subsequently, they were purified by passing through a 5-μm polycarbonate membrane filter before use to infect ICR mice.

### 2.2 Propagation of *T. Gondii* cysts in mice

Female ICR mice (6–8 weeks old) were obtained and housed under specific pathogen-free (SPF) conditions at a controlled temperature (23 ± 2 °C) and humidity (45 ± 10%) with a 12 h light/dark cycle. Mice had free access to irradiated chow and autoclaved water. Mice were inoculated intraperitoneally with 200 tachyzoites of the *T. gondii* ME49 strain in 200 μL sterile PBS using a 27-gauge needle. Body weight and general health were monitored daily. Brain tissues were aseptically collected, homogenized in sterile PBS, and examined under a phase-contrast microscope to count tissue cysts (Wang and Sibley, 2020). The harvested cysts were subjected to defined heat treatments before being used to orally infect C57BL/6J mice.

### 2.3 *T. Gondii* cysts heat treatment and mouse infection

Brain tissues were aseptically collected from chronically infected ICR mice (30 days post-infection) following euthanasia. Tissue homogenates were prepared in sterile normal saline, and *T. gondii* cysts were enumerated microscopically. The cyst concentration was adjusted to approximately 1 cyst per microliter. Aliquots of the homogenate were subjected to heat treatment in a metal heating block at temperatures of 45, 50, 55, 60, 65, and 70 °C for 10, 20, and 30 min. Subsequently, C57BL/6J mice (female, 6–8 weeks old) were orally infected via gavage with 200 μL of the heat-treated homogenate. For each combination of temperature and time point, five mice were used as biological replicates.

### 2.4 Routine observation and euthanasia sampling analysis of mice

Mice were observed daily for 20 days post-infection. Body weight was measured, and general conditions, including activity, food/water intake, and clinical signs, were monitored. At the endpoint (20 days post-infection or earlier if criteria were met), mice were euthanized. Blood samples were collected for serum isolation, and brain tissues were aseptically harvested. Serum was used to detect anti-*T. gondii* IgG antibodies via ELISA. Brain tissues were divided for subsequent qPCR analysis targeting the *T. gondii* B1 gene and for histopathological examination using hematoxylin and eosin (H&E) staining.

### 2.5 Spleen index and serum *T. Gondii*-specific antibodies

Following euthanasia, the body and spleen weights of the mice were measured to calculate the splenic index (mg/g). Terminal blood samples were collected via retro-orbital bleeding under isoflurane anesthesia at 20 days post-infection. Serum was separated by allowing blood to clot at room temperature for 30 min, followed by centrifugation at 2000 ×*g* for 15 min at 4 °C, and stored at −80 °C. Anti-*T. gondii* IgG antibody levels were quantified in duplicate using a commercial ELISA kit (VRL Asia Biotechnology, #VRL-ELISA-2023), with optical density read on a BioTek Synergy H1 microplate reader. Samples with an OD ≥ 0.5 were considered seropositive (Li et al., 2025; Sharma et al., 2019).

### 2.6 Detection of *T. Gondii* by real-time quantitative PCR (q-PCR)

To detect *T. gondii* DNA in mouse brain tissue, genomic DNA was extracted from brain homogenates collected at 20 days post-infection (Juránková et al., 2014). The DNA was subjected to qPCR targeting the *T. gondii* B1 gene (Lin et al., 2000; Opsteegh et al., 2010). A Ct value ≤35 was defined as positive for this study, while a Ct value >35 was considered negative. The Ct values and a standard curve were used to analyze the parasite DNA load following different heat treatments.

### 2.7 Histopathological analysis

Brain tissues were collected at 20 days post-infection and fixed in 4% neutral-buffered formalin. Subsequently, the tissues were processed using standard histological techniques, embedded in paraffin, and sectioned at 5 μm. Sections were stained with hematoxylin and eosin (H&E). Histopathological examination was performed using brightfield microscopy. The evaluation was conducted in accordance with established protocols (Dong et al., 2019).

### 2.8 Statistical analysis

Statistical analysis was performed using GraphPad Prism 10 (version 10.1.2). Data are presented as the mean ± SEM of three independent biological replicates. Body weight data were analyzed via two-way repeated-measures ANOVA. Survival curves were generated using the Kaplan-Meier method and compared by the log-rank (Mantel-Cox) test. Comparisons between two groups were conducted using an unpaired Student's *t*-test (parametric) or the Mann-Whitney *U* test (non-parametric), as determined by the normality and homogeneity of variance. A *P* value of less than 0.05 was considered statistically significant (*P < 0.05, **P < 0.01, ***P < 0.001, ****P < 0.0001; ns, not significant).

## 3 Results

### 3.1 Body weight changes and survival analysis of mice infected with heat-treated *T. Gondii* cysts

To determine the effect of heat treatment on the infectivity of cysts, tissue homogenates containing *T. gondii* cysts were treated at temperatures ranging from 45 °C to 70 °C (increments of 5 °C) for durations of 10, 20, and 30 min prior to being utilized for the oral infection of C57BL/6J mice.

**Fig. 1:**
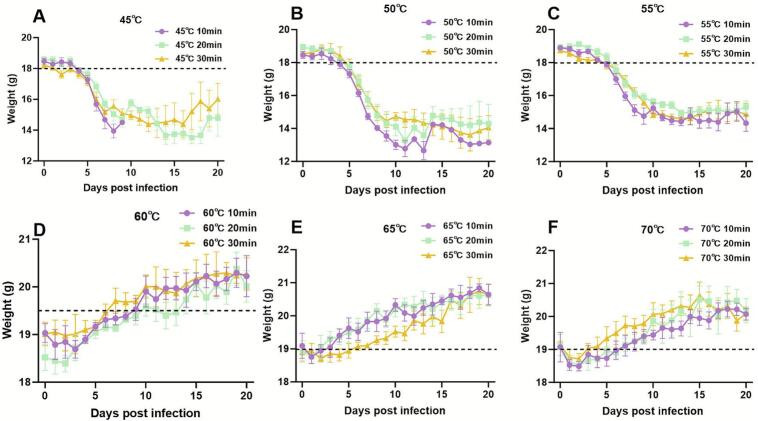
Body weight changes in mice infected with heat-treated *T. gondii* cysts. C57BL/6 J mice were orally infected with cysts treated at the indicated temperatures (ranging from 45 to 70 °C) and durations (10, 20, or 30 min). The body weight of the mice was monitored for 20 days after infection. Panels show curves for cysts treated at 45 °C (A), 50 °C (B), 55 °C (C), 60 °C (D), 65 °C (E), and 70 °C (F).

Changes in mouse body weight were monitored for 20 days post-infection (Fig. 1). Mice infected with cysts treated at 45 °C, 50 °C, or 55 °C for any of the specified durations (10, 20, or 30 min) showed a substantial decrease in body weight within the first week (Fig. 1A–C). The weight loss was less pronounced at 55 °C compared to lower temperatures. Conversely, no significant reduction in body weight was detected in mice infected with cysts treated at 60 °C, 65 °C, or 70 °C across all treatment times (Fig. 1D–F).

**Fig. 2:**
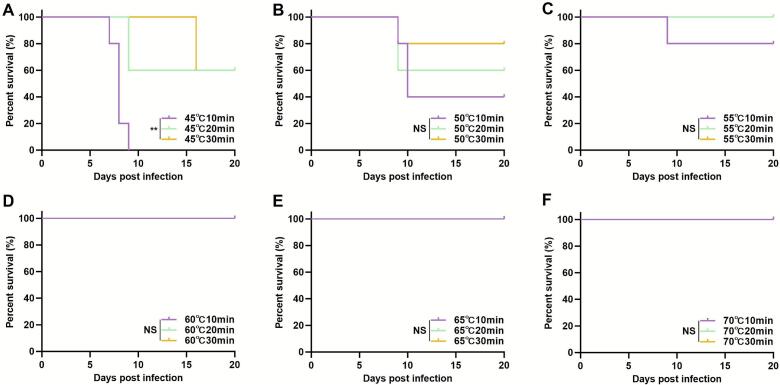
Survival of mice infected with heat-treated *T. gondii* cysts. Kaplan-Meier survival curves of C57BL/6 J mice following oral infection with cysts treated at 45 °C (A), 50 °C (B), 55 °C (C), 60 °C (D), 65 °C (E), and 70 °C (F) for 10, 20, and 30 min.

Survival was also monitored (Fig. 2). All mice infected with cysts that were treated at 45 °C for 10 min died. In contrast, the mortality rates were approximately 60% for the groups treated at this temperature for 20 and 30 min, respectively (Fig. 2A). At 50 °C, mortality rates were 40% (10 min), 60% (20 min), and 80% (30 min) (Fig. 2B). At 55 °C, deaths were only observed in the 10-min treatment group, and all mice in the 20- and 30-min treatment groups survived (Fig. 2C). Crucially, all mice survived following infection with cysts treated at 60 °C, 65 °C, and 70 °C for all durations (10, 20, and 30 min) (Fig. 2D–F).

### 3.2 Splenic response and seroconversion following infection with heat-treated cysts

**Fig. 3:**
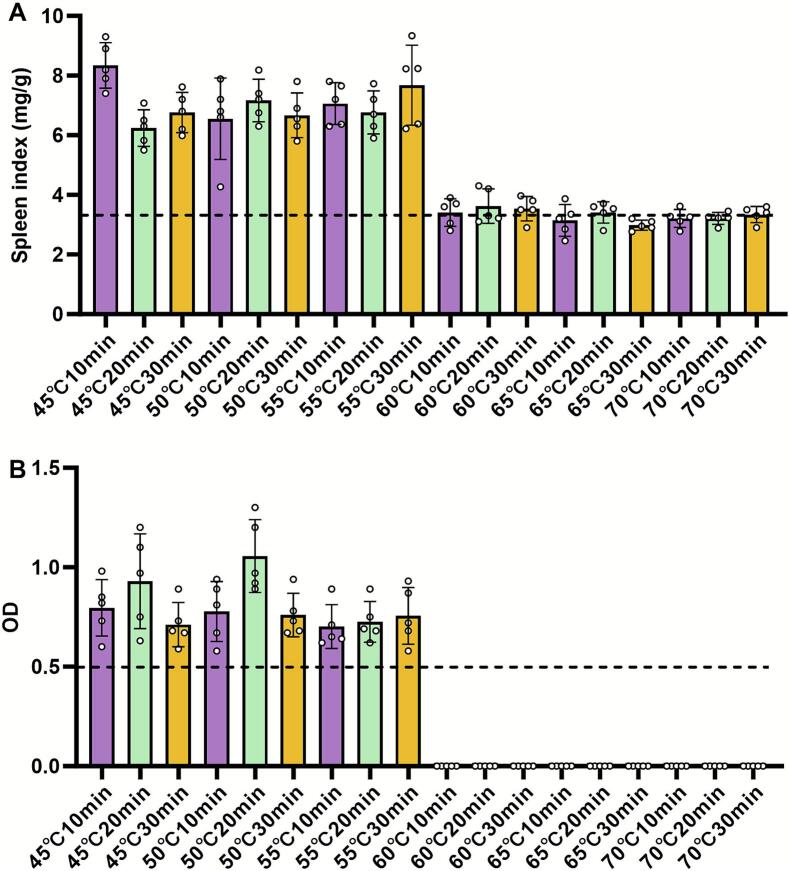
Splenic index and serological response in mice infected with heat-treated cysts. (A) Splenic index (spleen weight/body weight) of mice at 20 days after infection. (B) Optical density (OD) values of serum anti-*T. gondii* IgG antibodies measured by ELISA. Data are obtained from infection with cysts treated at 45, 50, 55, 60, 65, and 70 °C for the indicated durations. The dashed line in (B) indicates the positive cutoff (OD = 0.5).

The splenic index and serological status of mice infected with heat-treated *T. gondii* cysts were assessed to further evaluate infectivity (Fig. 3).

Mice infected with cysts that were treated at 45 °C, 50 °C, or 55 °C for any duration (10, 20, or 30 min) developed significant splenomegaly, with splenic indices all exceeding 3.5. Notably, the most pronounced enlargement was observed in the 45 °C (10 min) and 55 °C (30 min) groups. In contrast, infection with cysts treated at 60 °C, 65 °C, or 70 °C for all durations did not lead to significant splenomegaly, and the splenic indices remained at the baseline levels (approximately 3.5 ± 0.2) (Fig. 3A).

Consistent with the splenic response, serum anti-*T. gondii* IgG antibodies were detected (OD ≥ 0.5, considered positive) in all mice infected with cysts that were treated at 45 °C, 50 °C, or 55 °C. Conversely, mice infected with cysts treated at 60 °C, 65 °C, or 70 °C for any duration were seronegative, with OD values below the 0.5 threshold (Fig. 3B). These results indicate the absence of systemic infection in these groups.

### 3.3 Detection of *T. Gondii* DNA in brain tissue by qPCR

**Fig. 4:**
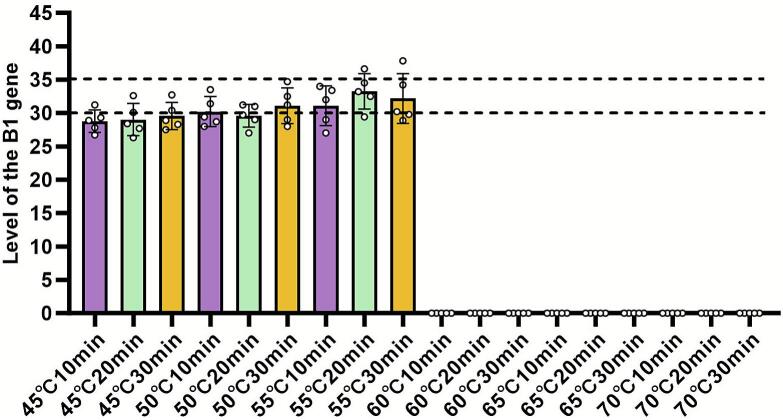
Detection of *T. gondii* DNA in brain tissue by qPCR. Parasite burden in the brain was quantified by qPCR targeting the *T. gondii* B1 gene 20 days after infection. Ct values are shown for mice infected with cysts treated at the indicated temperatures and durations. Ct ≤ 35 (dashed line) was considered positive for parasite DNA.

The presence of *T. gondii* DNA in the brain tissue of infected mice was assessed by qPCR targeting the parasite B1 gene (Fig. 4). A high parasite DNA load (indicated by Ct values ≤35) was detected in the brains of all mice infected with cysts treated at 45 °C, 50 °C, or 55 °C, regardless of treatment duration (10, 20, or 30 min). This validates the successful colonization of the parasite following these sub-inactivating heat treatments. In stark contrast, no specific *T. gondii* DNA signal (Ct > 35) was detected in any mouse infected with cysts treated at 60 °C, 65 °C, or 70 °C for any duration. These results provide molecular evidence that the heat treatments at or above 60 °C completely prevented the establishment of the parasite in the host brain.

### 3.4 Histological analysis of brain tissue in mice

**Fig. 5:**
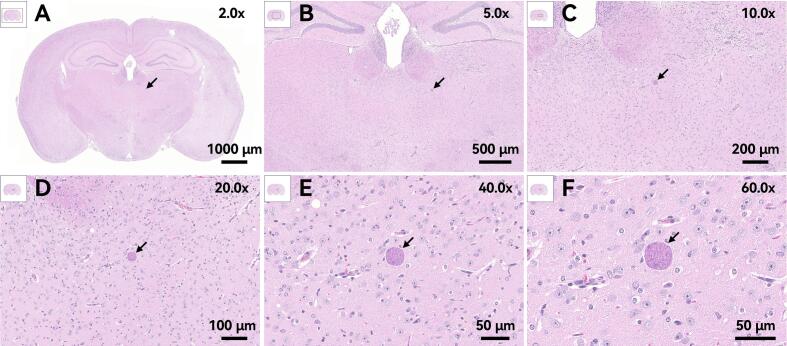
Histopathological analysis of brain tissue from mice infected with heat-treated cysts. Representative H&*E*-stained brain sections from a mouse infected with cysts treated at 50 °C for 10 min are presented. Images were acquired at magnifications of ×2.0 (A), ×5.0 (B), ×10.0 (C), ×20.0 (D), ×40.0 (E), and ×60.0 (F). Arrows indicate *T. gondii* tissue cysts. No cysts or lesions were observed in any brain sections from mice infected with cysts treated at ≥60 °C.

A histopathological examination of the brain tissue was carried out to visually evaluate parasite colonization (Fig. 5). In mice infected with cysts treated at sub-inactivating temperatures (45 °C, 50 °C, or 55 °C), characteristic *T. gondii* tissue cysts were readily observed in H&*E*-stained brain sections at 20 days after infection (representative images from the 50 °C, 10-min group are shown in Fig. 5A–F). This validates the successful invasion and establishment of infection in the central nervous system following these treatments. Conversely, when all other parameters were in full agreement, brain tissues from mice infected with cysts that had been treated at 60 °C, 65 °C, or 70 °C were completely devoid of any *T. gondii* cysts or associated pathological lesions. This histological evidence definitively supports the conclusion that thermal treatment at or above 60 °C fully inactivates the cysts and prevents neuroinvasion.

## 4 Discussion

Our integrated bioassay demonstrates that heating *T. gondii* cysts to 60 °C for 10 min represents a critical threshold that is sufficient to eliminate infectivity in susceptible mice. This finding refines the thermal inactivation parameters for tissue cysts and is directly validated by a comprehensive panel of endpoints: 100% survival, absence of seroconversion, undetectable parasite DNA in the brain, and no histopathological evidence of infection.

This determined threshold aligns with key precedents in the literature while highlighting the importance of experimental context. Early work indicated that cysts can be inactivated at 60 °C for 10–15 min, and a more recent risk-assessment model validates 64 °C as a safe internal temperature for meat (Rani and Pradhan, 2021). The subtle variations among studies, including those reported in our study, presumably originate from significant methodological disparities. These include the characteristics of the parasite material (e.g., tachyzoites in milk versus tissue cysts) (Khosravi et al., 2025), the heat transfer medium (solid meat matrix versus tissue homogenate) (Dubey et al., 1990), and the sensitivity of the bioassay host (Dubey et al., 1970). Such factors underscore the persistent challenge in the field of the lack of standardized, validated detection methods for *T. gondii* in food products (Bouwknegt et al., 2018; Gomez-Samblas et al., 2016).

Our study directly addresses the requirement for a definitive infectivity assessment. While molecular techniques (Kim et al., 2024) and in vitro culture (Dawant et al., 2024; Opsteegh et al., 2020) offer speed and scalability for screening, the mouse bioassay remains the indispensable gold standard for confirming true viability and public health risk (Opsteegh et al., 2020). The high global seroprevalence in livestock (Gamble, et al., 2019; Hajimohammadi et al., 2022), such as 19% in pigs (Foroutan et al., 2019), 29% in pigs in China (Zhang et al., 2019), and approximately 33% in sheep and goats (Ahaduzzaman and Hasan, 2022), coupled with the dominant role of foodborne transmission (Shapiro et al., 2019), makes such a reliable assessment of utmost importance. Therefore, our work provides a robust experimental benchmark with clear dual applications. In the context of laboratory biosafety, it provides a validated protocol for inactivating mouse tissues infected with cysts. For public health and food safety, it reinforces practical guidance: cooking meat to ensure a core temperature of at least 60–65 °C and maintaining it for several minutes is an effective and necessary measure to mitigate risk (Kotula et al., 1991; Rani and Pradhan, 2021).

Looking ahead, effective control of this zoonotic threat (Almeria and Dubey, 2021) will require coordinated advances on multiple fronts. First, a deeper investigation into the structural and metabolic determinants of cyst thermotolerance (Christiansen et al., 2022; Kumar et al., 2024; Waldman et al., 2020) is essential for informing more targeted and reliable inactivation strategies. Second, developing and optimizing novel technologies (e.g., microwave and pulsed heat) and combined physico-chemical hurdles (Anumudu et al., 2024; Barbhuiya et al., 2021) could enhance efficiency while preserving food quality. Third, parallel efforts are required to establish highly sensitive, standardized direct-detection methods, potentially integrating molecular viability markers (Wu et al., 2024), to complement biological assays. Through such multidisciplinary integration, a more precise and effective system for managing *T. gondii* risks can be achieved, ultimately strengthening food safety and public health protection.

## 5 Conclusions

This study conclusively demonstrates that thermal treatment at 60 °C for 10 min completely inactivates *T. gondii* tissue cysts, as robustly confirmed by a multi-parameter assessment including survival, serology, qPCR, and histopathology. The findings provide a precise benchmark for laboratory biosafety protocols and present a science-based parameter to inform thermal processing guidelines for meat, directly contributing to the prevention of foodborne toxoplasmosis. Future research should focus on the mechanisms of cyst thermotolerance and the development of effective inactivation methods that also preserve food quality.

## CRediT authorship contribution statement

**Zhao Li:** Writing – review & editing, Writing – original draft, Visualization, Validation, Supervision, Software, Project administration, Investigation, Funding acquisition, Formal analysis, Data curation, Conceptualization. **Tao Li:** Methodology, Data curation. **Lian-Tao Yang:** Methodology, Data curation, Conceptualization. **Cai-Qin Deng:** Methodology, Data curation. **Qi-Xin Liu:** Methodology. **Qin-Zhang:** Methodology. **Ling Wu:** Visualization, Software. **Yue Sun:** Methodology. **Feng-Cai Zou:** Conceptualization, Supervision. **Xue Zhou:** Visualization, Software, Methodology. **Qi-Shuai Liu:** Supervision, Resources, Funding acquisition, Conceptualization.

## Ethics statement

All experiments involving mice were conducted in strict accordance with the Guidelines for the Management and Use of Laboratory Animals issued by the Animal Research and Resource Center of Yunnan University (CNAS LA0029). The animal protocol and ethical aspects of the study were reviewed and approved by the Institutional Animal Care and Use Committee (IACUC) of Yunnan University (Approval No. YNU20241024).

## Declaration of competing interest

The authors declare that they have no competing interests.
